# An interplay between UCP2 and ROS protects cells from high-salt-induced injury through autophagy stimulation

**DOI:** 10.1038/s41419-021-04188-4

**Published:** 2021-10-08

**Authors:** Maurizio Forte, Franca Bianchi, Maria Cotugno, Simona Marchitti, Rosita Stanzione, Vittorio Maglione, Sebastiano Sciarretta, Valentina Valenti, Roberto Carnevale, Francesco Versaci, Giacomo Frati, Massimo Volpe, Speranza Rubattu

**Affiliations:** 1 IRCCS Neuromed, Pozzilli, Isernia Italy; 2 Department of Medical-Surgical Sciences and Biotechnologies, Sapienza University of Rome, Latina, Italy; 3 Division of Cardiology, Santa Maria Goretti Hospital, Latina, Italy; 4 Mediterranea Cardiocentro, Naples, Italy; 5 Department of Clinical and Molecular Medicine, Sapienza University of Rome, Rome, Italy

**Keywords:** Macroautophagy, Macroautophagy

## Abstract

The mitochondrial uncoupling protein 2 (UCP2) plays a protective function in the vascular disease of both animal models and humans. UCP2 downregulation upon high-salt feeding favors vascular dysfunction in knock-out mice, and accelerates cerebrovascular and renal damage in the stroke-prone spontaneously hypertensive rat. Overexpression of UCP2 counteracts the negative effects of high-salt feeding in both animal models. We tested in vitro the ability of UCP2 to stimulate autophagy and mitophagy as a mechanism mediating its protective effects upon high-salt exposure in endothelial and renal tubular cells. UCP2 silencing reduced autophagy and mitophagy, whereas the opposite was true upon UCP2 overexpression. High-salt exposure increased level of reactive oxygen species (ROS), UCP2, autophagy and autophagic flux in both endothelial and renal tubular cells. In contrast, high-salt was unable to induce autophagy and autophagic flux in UCP2-silenced cells, concomitantly with excessive ROS accumulation. The addition of an autophagy inducer, Tat-Beclin 1, rescued the viability of UCP2-silenced cells even when exposed to high-salt. In summary, UCP2 mediated the interaction between high-salt-induced oxidative stress and autophagy to preserve viability of both endothelial and renal tubular cells. In the presence of excessive ROS accumulation (achieved upon UCP2 silencing and high-salt exposure of silenced cells) autophagy was turned off. In this condition, an exogenous autophagy inducer rescued the cellular damage induced by excess ROS level. Our data confirm the protective role of UCP2 toward high-salt-induced vascular and renal injury, and they underscore the role of autophagy/mitophagy as a mechanism counteracting the high-salt-induced oxidative stress damage.

## Introduction

Cardiovascular diseases represent a major health issue for which new preventive and therapeutic strategies are needed, mostly based on a deeper knowledge of the underlying pathogenic mechanisms. The molecular mechanisms involved in the cardiovascular damage are multiple, some of them being discovered through the use of appropriate animal models.

Among others, mitochondrial uncoupling protein 2 (UCP2) is an anion carrier located within the inner mitochondrial membrane and involved in the regulation of energy production, cellular homeostasis, oxidative stress, and cell survival [1, 2]. We previously demonstrated that UCP2 is downregulated by ageing and by high-salt feeding in the animal model of stroke-prone spontaneously hypertensive rat (SHRSP), promoting renal and cerebrovascular damage in this model [3–7]. On the other hand, UCP2 upregulation could rescue cell viability, reduce vascular damage and stroke occurrence in the high-salt fed SHRSP [4, 6, 8]. Similarly, lack of UCP2 favored vascular dysfunction in mice exposed to high-salt diet independent of blood pressure levels [9]. In this experimental setting, UCP2 upregulation effectively counteracted the vascular dysfunction [10].

Based on the experimental evidence, UCP2 upregulation exerts protective effects by reducing oxidative stress, inflammation and the consequent cellular damage. On the other hand, UCP2 downregulation is detrimental as it is associated with excessive reactive oxygen species (ROS) accumulation, consequent damage of macromolecules (DNA, lipids, proteins) and cell death. Therefore, the anti-oxidant and anti-inflammatory properties appear the most relevant mechanisms mediating the beneficial effects of UCP2. Of importance, UCP2 turns out to be a promising therapeutic target in cardiovascular diseases [1, 2, 11, 12].

From this perspective, it would be important to broad our knowledge by investigating the fine mechanisms of action of UCP2. In this regard, a link between UCP2 protection and autophagy stimulation has recently emerged with particular regard to cardiomyopathies, including ischemia/reperfusion (I/R) injury and cardiac hypertrophy, acute kidney injury and cerebral ischemia [13–18].

In the present work, we investigated the relationship between high-salt-induced injury, UCP2 protection and autophagy stimulation by using cerebral endothelial and renal epithelial tubular cell lines. We demonstrate that UCP2 protects both endothelial and renal tubular cells from high-salt-induced damage through autophagy stimulation, and that mitophagy is also regulated by UCP2. Our findings indicate that ROS mediate the link between UCP2 and autophagy.

## Results

### UCP2 modulates autophagy

**Fig. 1:**
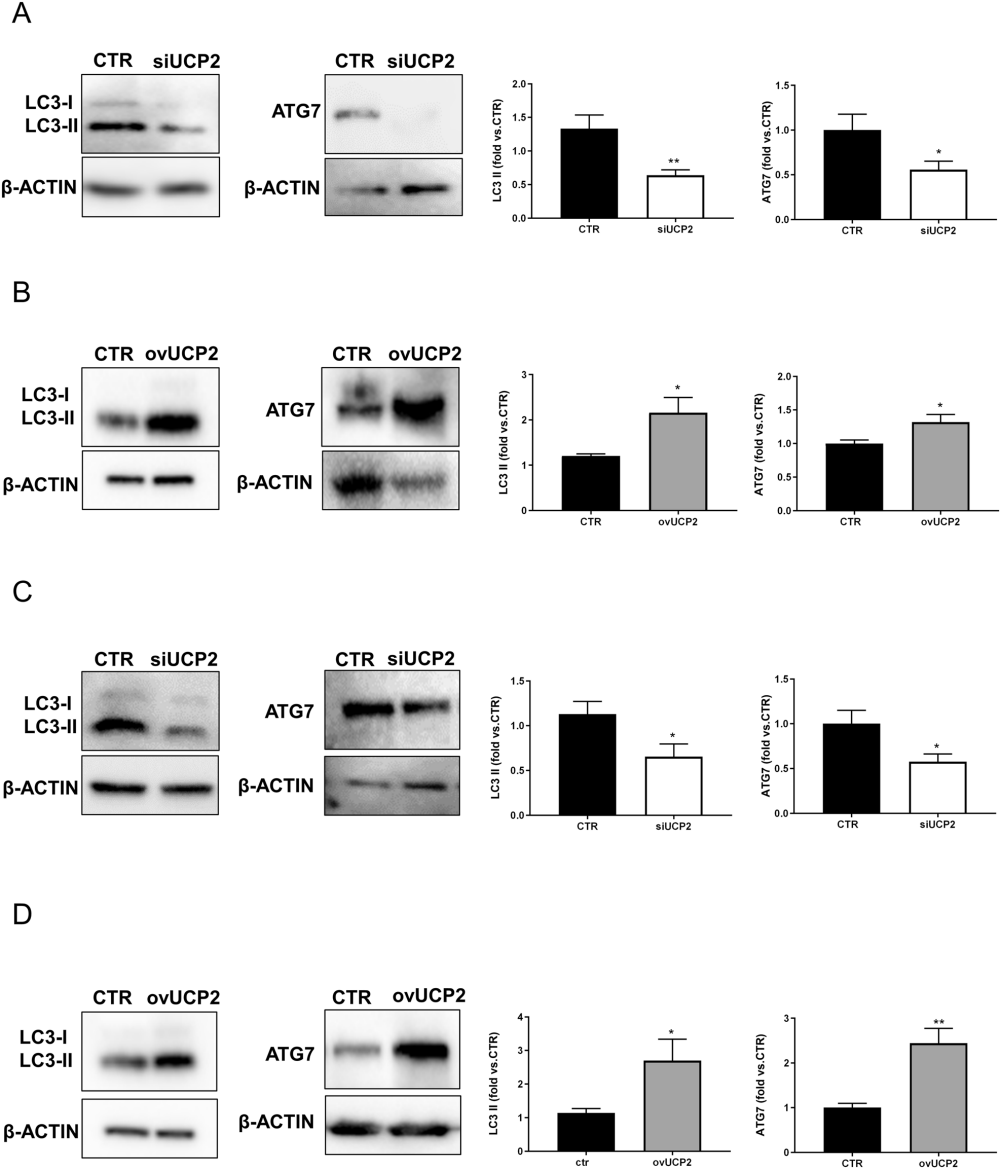
UCP2 downregulation reduces autophagy whereas UCP2 overexpression is associated with increased autophagy. Level of autophagy in UCP2-silenced microvascular endothelial cells (siUCP2) (**A**) or cells overexpressing UCP2 (ovUCP2) for 24 h (*N* = 5) (**B**). Level of autophagy in renal proximal tubular epithelial cells silenced for UCP2 (siUCP2) (**C**) or overexpressing UCP2 (ovUCP2) for 24 h (**D**) (*N* = 7). CTR indicates not transfected cells. Representative western blots of LC3-I/II and ATG7 and corresponding quantification are reported. *p < 0.05, **p < 0.001 obtained using the two-tailed students *t*-test. Data are reported as mean ± SEM.

In order to assess whether UCP2 was associated with autophagy, we analyzed the expression of two known markers of autophagy (LC3 and ATG7) in cells lacking or overexpressing UCP2. The Fig. 1 shows that UCP2 silencing reduced LC3-II and Atg7 expression in both cell lines (Fig. 1A, C). Consistently, the UCP2 overexpression increased the autophagy markers in both cell lines (Fig. 1B, D). This evidence demonstrates that UCP2 tightly interacts with autophagy. The rate of both UCP2 silencing and overexpression is reported in the Supplementary Fig. 1A, B.

### High-salt stimulates UCP2 expression and autophagy activation whereas UCP2 silencing blunts the autophagy response to high-salt

**Fig. 2:**
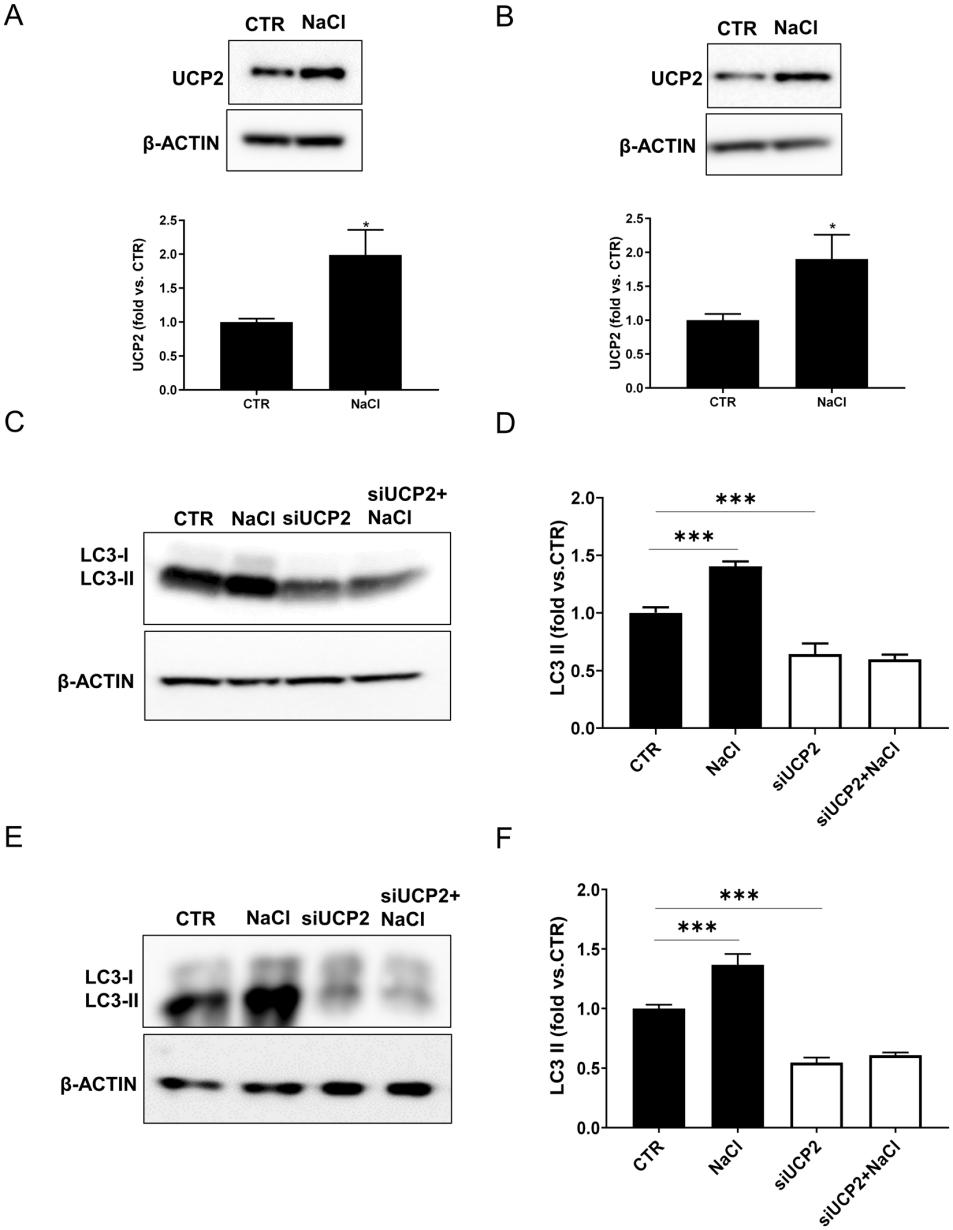
High-salt treatment enhances UCP2 expression along with autophagy activation. Level of UCP2 in microvascular endothelial cells (**A**) and in renal proximal tubular epithelial cells (**B**) treated for 72 h with 20 mM NaCl (*N* = 6). Representative western blots of UCP2 and corresponding quantification are shown. *p < 0.05, **p < 0.001 obtained using the two-tailed students *t*-test. **C**–**F** Level of autophagy in microvascular endothelial cells (**C**, **D**) and in renal proximal tubular epithelial cells (**E**, **F**) silenced for UCP2 (siUCP2) and treated for 72 h with 20 mM NaCl (*N* = 4). Representative western blots of LC3-I/II and ATG7 and corresponding quantification are reported. CTR indicates not treated cells and not transfected cells. ***p < 0.001 obtained using the one-way ANOVA followed by Bonferroni post hoc analysis. Data are reported as mean ± SEM.

**Fig. 3:**
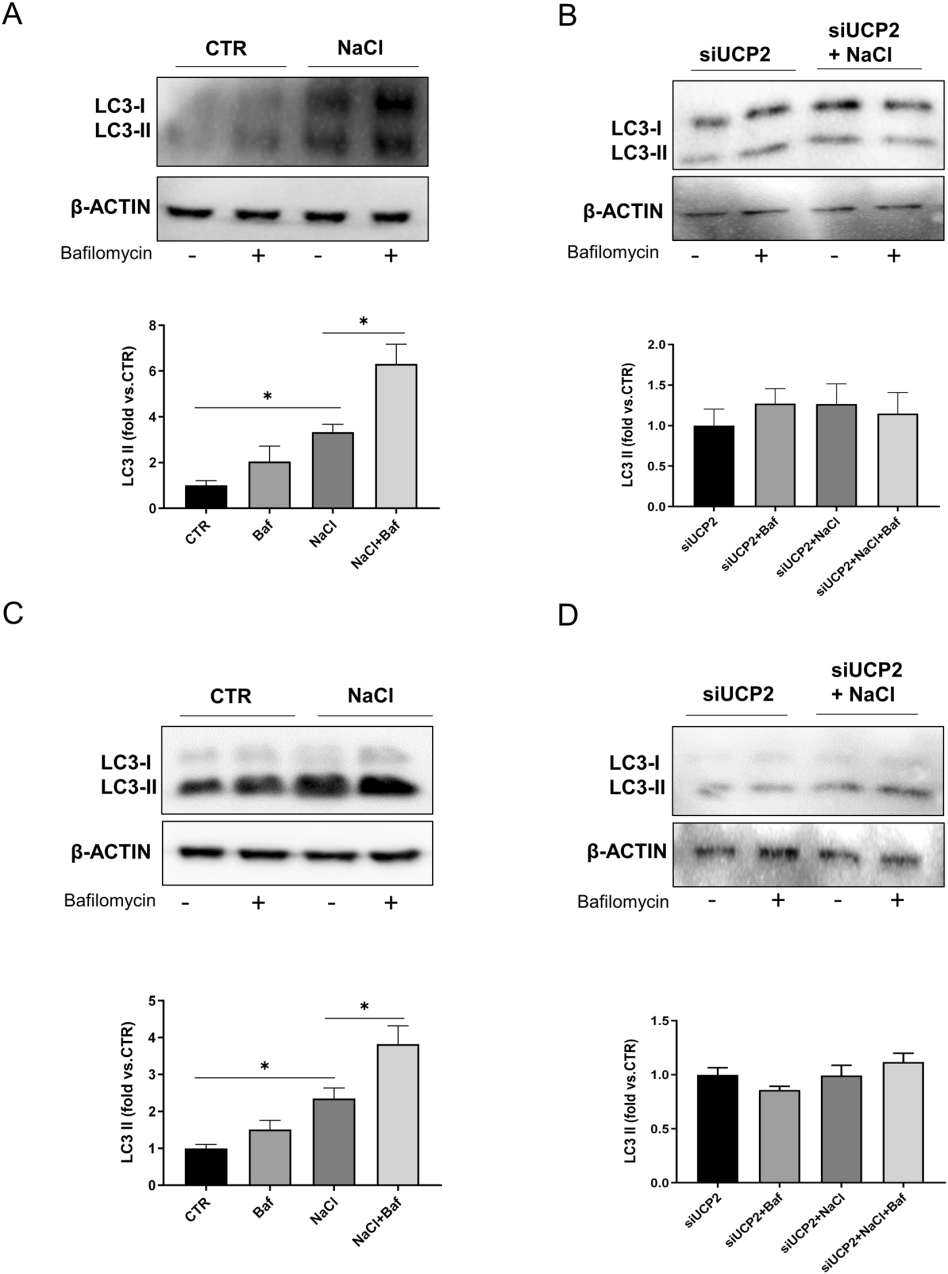
Autophagic flux evaluation in UCP2-silenced cells exposed to high-salt. **A** Autophagic flux evaluation in microvascular brain endothelial cells exposed to high-salt (20 mM NaCl for 72 h) and treated for the last 3 h with 100 nM bafilomycin (*N* = 4). **B** Autophagic flux evaluation in UCP2-silenced endothelial cells (siUCP2) exposed to high-salt (20 mM NaCl for 72 h) and treated for the last 3 h with 100 nM bafilomycin (*N* = 4). **C** Autophagic flux evaluation in renal proximal tubular epithelial cells exposed to high-salt (20 mM NaCl for 72 h) and treated for the last 3 h with 100 nM bafilomycin (*N* = 4). **D** Autophagic flux evaluation in UCP2-silenced renal proximal tubular epithelial cells (siUCP2) exposed to high-salt (20 mM NaCl for 72 h) and treated for the last 3 h with 100 nM bafilomycin (*N* = 4). Representative western blots for LC3-I/II and corresponding quantification are reported. *p < 0.05, obtained using the one-way ANOVA followed by Bonferroni post hoc analysis. Data are reported as mean ± SEM.

**Fig. 4:**
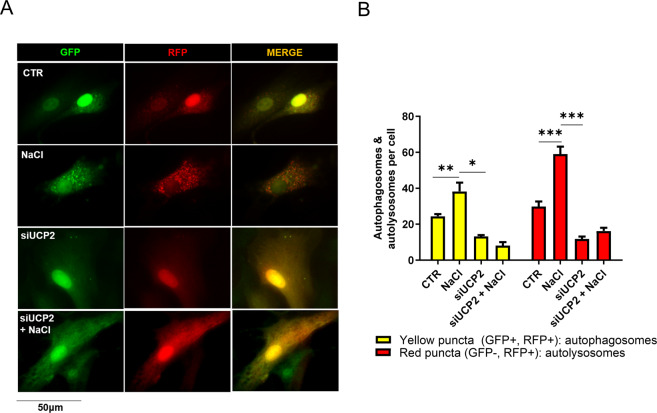
Autophagic flux evaluation by the mRFP-GFP-LC3 tandem probe in UCP2-silenced cells exposed to high-salt. **A**, **B** High-salt treated siUCP2 microvascular brain endothelial cells were transduced with adenovirus overexpressing mRFP-GFP-LC3 to evaluate LC3 puncta. Yellow dots (merged red and green) indicate autophagosomes whereas red dots indicate autolysosomes. Representative images (**A**) and corresponding quantification (**B**). (*N* = 5). *p < 0.05, **p < 0.01, ***p < 0.001 obtained using the one-way ANOVA followed by Bonferroni post hoc analysis. Data are reported as mean ± SEM.

In order to explore whether UCP2 was associated with stress-induced autophagy, we exposed cells to high-salt treatment. First, we observed the increase of UCP2 expression in both endothelial and epithelial tubular cells exposed to high-salt (Fig. 2A, B). In the same condition, autophagy was activated as documented by the increased expression of LC3-II (Fig. 2C–F). This evidence is consistent with a self-defensive response of endothelial and renal tubular cells against high-salt injury through UCP2 and autophagy stimulation. In fact, we further showed that, when the high-salt exposure was performed in the absence of UCP2, LC3-II failed to rise in both cell lines (Fig. 2C–F). Of note, UCP2 silencing lowered the baseline expression of the autophagy markers in both cell lines **(**Fig. 2C–F). Next, we further analyzed the autophagic flux by using bafilomycin as an inhibitor of lysosome degradation and also in cells with adenoviral-mediated overexpression of mRFP-GFP-LC3. We observed an increase of LC3-II in both cell lines upon high-salt and a further increase in the presence of bafilomycin, suggesting an increased autophagic flux following high-salt treatment (Fig. 3A, C). Conversely, in UCP2-silenced cells LC3-II did not respond to bafilomycin treatment, suggesting that autophagy and autophagosome formation were suppressed in these conditions (Fig. 3B, D). These results were corroborated by the observation that an increased number of both autophagosomes (yellow and red fluorescent dots) and autolysosomes (red dots only) was present in cells exposed to high-salt whereas only few dots were detected in UCP2-silenced cells (Fig. 4A, B).

This evidence demonstrates a fundamental role of UCP2 in the self-defensive response of endothelial and renal tubular cells against high-salt-induced injury through autophagy stimulation.

### UCP2 modulates mitophagy

**Fig. 5:**
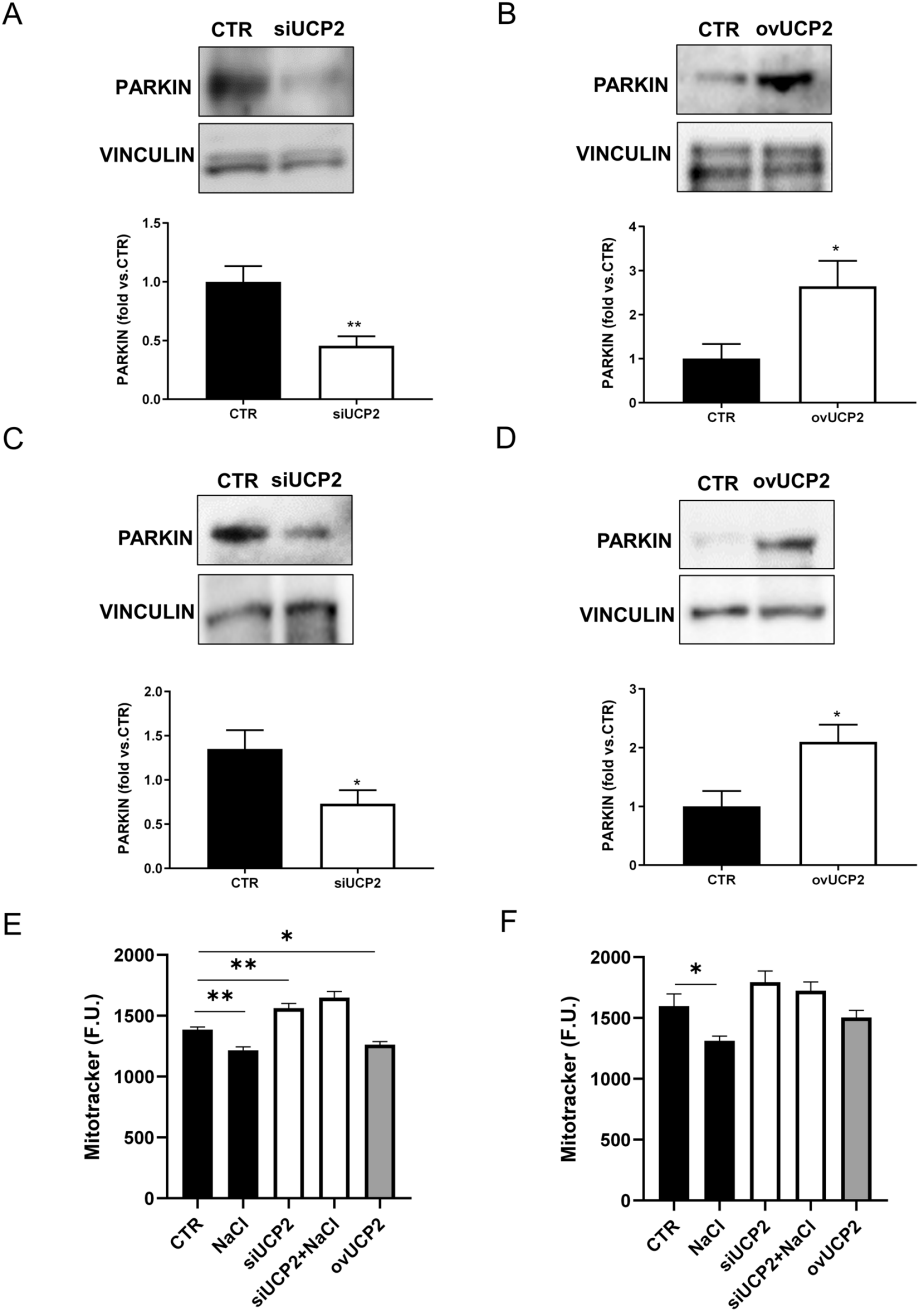
UCP2 modulates mitophagy. Level of PARKIN in UCP2-silenced microvascular endothelial cells (siUCP2) (**A**) or cells overexpressing UCP2 (ovUCP2) for 24 h (*N* = 4) (**B**). Level of PARKIN in renal proximal tubular epithelial cells silenced for UCP2 (siUCP2) (**C**) or overexpressing UCP2 (ovUCP2) for 24 h (**D**) (*N* = 4). CTR indicates not transfected cells. Representative western blots of PARKIN and corresponding quantification are reported. *p < 0.05, **p < 0.001 obtained using the two-tailed students *t*-test. **E**, **F** Analysis of mitophagy through MitoTracker green (F.U., fluorescent units) in high-salt- treated siUCP2 or ovUCP2 microvascular endothelial cells (**E**) and renal proximal tubular epithelial cells (**F**) (*N* = 12). *p < 0.05, **p < 0.001 obtained using the one-way ANOVA followed by Bonferroni post hoc analysis. Data are reported as mean ± SEM.

Since UCP2 plays an important role in ensuring mitochondrial homeostasis [1, 2] we investigated whether mitophagy, the selective form of autophagy for mitochondria [19], was altered in UCP2-silenced cells. As shown in Fig. 5, PARKIN, a well-known marker of mitophagy [19], was downregulated in both cell lines when silenced for UCP2 (Fig. 5A, C). In contrast, PARKIN was upregulated in cells overexpressing UCP2 (Fig. 5B, D). We also evaluated mitochondrial mass as an indirect marker of mitophagy by means of the fluorescent probe MitoTracker green. We found a substantial increase of MitoTracker signal in UCP2-silenced cells, which directly correlates with the number of mitochondria [20]. In contrast, high-salt exposure of not silenced cells led to a decrease of mitochondrial mass. A slight reduction was also observed in cells overexpressing UCP2 (Fig. 5E, F). In order to exclude that the increase of mitochondrial mass in UCP2-silenced cells could be attributable to the modulation of mitochondrial biogenesis, we evaluated the expression levels of PGC1α, the main regulator of mitochondrial biogenesis. We did not observe any difference between UCP2-silenced cells and those overexpressing UCP2 (Supplementary Fig. 2A, B).

These results suggest that UCP2 knockdown leads to mitophagy reduction, which in turn contributes to the accumulation of defective mitochondria, and that this process takes place through a PARKIN-dependent mechanism.

### UCP2-induced autophagy is critical for the regulation of cellular viability, apoptosis, and necrosis

**Fig. 6:**
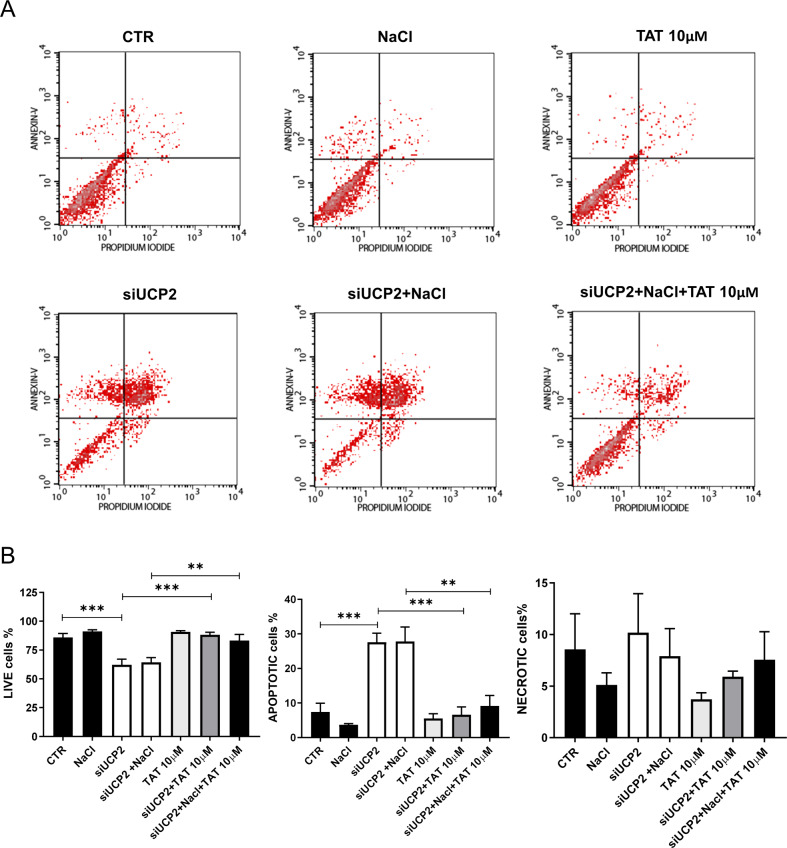
Reactivation of autophagy rescues cell death in UCP2-silenced endothelial cells. FACS analysis of microvascular endothelial cells silenced for UCP2 (siUCP2) and treated for 72 h with 20 mM NaCl either in the presence or in the absence of TAT-Beclin D11 (10 μM) (*N* = 6). Representative scatter plots (**A**) and quantification of live, apoptotic, and necrotic cells (**B**) are reported. CTR indicates not transfected and not treated cells. **p < 0.01, ***p < 0.001 obtained using the one-way ANOVA followed by Bonferroni post hoc analysis. Data are reported as mean ± SEM.

**Fig. 7:**
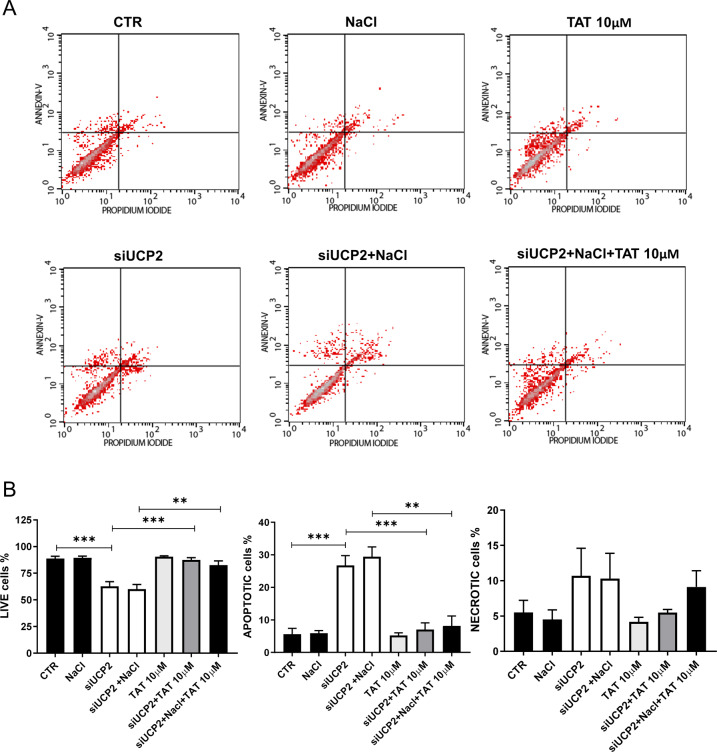
Reactivation of autophagy rescues cell death in UCP2-silenced proximal tubular epithelial cells. FACS analysis of renal proximal tubular epithelial cells silenced for UCP2 (siUCP2) and treated for 72 h with 20 mM NaCl either in the presence or in the absence of TAT-Beclin D11 (10 μM) (*N* = 6). Representative scatter plots (**A**) and quantification of live, apoptotic, and necrotic cells (**B**) are reported. CTR indicates not transfected and not treated cells. **p < 0.01, ***p < 0.001 obtained using the one-way ANOVA followed by Bonferroni post hoc analysis. Data are reported as mean ± SEM.

In order to gain deeper insights on the role of UCP2 through autophagy stimulation on cell viability, particularly when cells are exposed to high-salt, we assessed the rate of cell viability, apoptosis and necrosis in both endothelial and renal tubular cells by cell sorting fluorescence analysis. Results are shown in Figs. 6 and 7. Cells exposed to high-salt did not substantially modify their rate of viability, apoptosis and necrosis. In contrast, UCP2 silencing significantly decreased cell viability and increased both apoptosis and necrosis rates, with no further changes upon high-salt exposure (Figs. 6A, B and 7A, B). The addition of TAT-Beclin 1, an autophagy stimulator [21], rescued cell viability and reduced the rate of both apoptosis and necrosis in UCP2-silenced cells even when exposed to high-salt (Figs. 6 and 7). Of note, we assessed that the amount of TAT-Beclin 1 needed in order to re-establish a normal condition in UCP2-silenced cells was higher when they were exposed to high-salt (data not shown). Altogether, our results demonstrate that cell viability is preserved, at least in part, through autophagy stimulation upon exposure to high-salt. Based on our findings, UCP2 stimulation mediates the autophagy activation in response to high-salt treatment. The key role of UCP2 in this process is further underscored by the evidence that its silencing associated with a marked reduction of cell viability and an increased rate of apoptosis and necrosis. In this condition, the tight link between UCP2 and autophagy is proven by the evidence that autophagy activation by TAT-Beclin 1 could preserve cell viability in the absence of UCP2 even when cells were exposed to high-salt. Thus, autophagy activation appears the most appropriate strategy to rescue cell viability and protects cells from the high-salt injury in the absence of UCP2.

### UCP2 mediates the link between ROS and autophagy upon high-salt condition

**Fig. 8:**
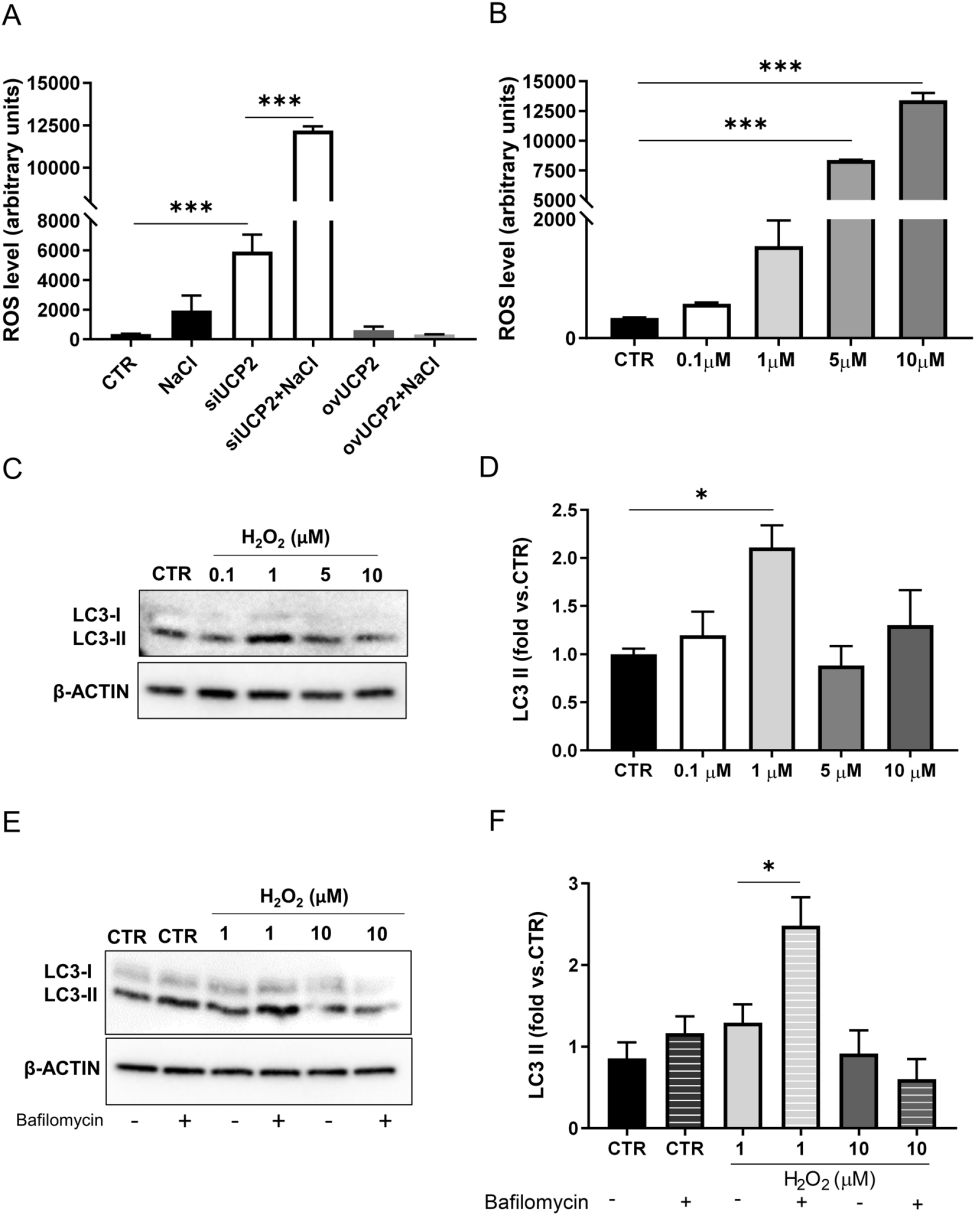
UCP2 gene silencing leads to ROS-induced inhibition of autophagy. **A** ROS levels were assessed in microvascular endothelial cells (RBMEC) silenced for 24 h for UCP2 (siUCP2) or transfected with a plasmid overexpressing UCP2 (ovUCP2), either in the presence or in the absence of high-salt treatment (NaCl 20 mM) for 72 h (*N* = 8) **B** ROS levels were assessed in RBMEC after treatment with different concentrations of H_2_O_2_ for 12 h (N = 3). **C**, **D** Evaluation of autophagy in RBMEC after treatment with different concentrations of H_2_O_2_ for 12 h. Representative western blot of LC3-I/II (**C**) and corresponding quantification (**D**) are shown (*N* = 6). **E**, **F** Autophagic flux evaluation in RBMEC after treatment with different concentrations of H_2_O_2_ for 12 h either in the presence or in the absence of 100 nM bafilomycin for the last 3 h. Representative western blot of LC3-I/II (**E**) and corresponding quantification (**F**) are shown (*N* = 6). CTR indicates not transfected and not treated cells. *p < 0.05, ***p < 0.001 obtained using the one-way ANOVA followed by Bonferroni post hoc analysis. Data are reported as mean ± SEM.

In a separate set of studies performed in the endothelial cell line we assessed the rate of ROS accumulation depending on the specific experimental condition. As shown in Fig. 8A, the level of ROS increased progressively from high-salt exposure to UCP2 silencing and reached a maximum level upon high-salt exposure of UCP2-silenced cells. On the other hand, the UCP2 overexpression markedly reduced ROS level. Therefore, ROS represent a damaging factor released in the presence of high-salt condition, particularly when UCP2 is lacking and high-salt is added. It is known that an excess of ROS can turn off autophagy [22]. To test the hypothesis that an excess of ROS could be responsible of the autophagy inhibition observed upon UCP2 silencing (see Fig. 2), we attempted to reproduce, through the use of H_2_O_2_, the same amount of ROS generated by lack of UCP2 and by salt exposure of UCP2-silenced cells. Through this approach, we assessed that the maximum amount of ROS generated in the presence of UCP2 silencing was comparable to that produced by 5 μM of H_2_O_2_ after 12 h of exposure, and that generated in UCP2-silenced cells exposed to high-salt was comparable to the amount produced by 10 μM H_2_O_2_ (Fig. 8A, B). The parallel assessment of the LC3-II expression level upon the different ROS concentrations generated by H_2_O_2_ demonstrated that the autophagy marker reached its maximum level at a ROS concentration generated by 1 μM of H_2_O_2_ (well below the ROS level generated by UCP2 silencing) (Fig. 8C, D). Afterwards, the autophagy marker did not increase (Fig. 8C, D). In this condition, we also analyzed the autophagic flux, as reported in the Fig. 8E, F. Thus, cells treated with 1 μM H_2_O_2_ displayed increased levels of LC3-II, which further increased in the presence of bafilomycin, whereas LC3-II did not respond to bafilomycin in cells treated with 10 μM H_2_O_2_, suggesting that autophagy is stimulated only by low ROS.

This evidence shows that the increase of ROS upon a stress such as high-salt exposure favors UCP2 expression with a consequent autophagy induction. However, when the accumulation of ROS reaches a very high level, such as that observed following UCP2 silencing, the autophagy machinery is no longer activated, not even by alternative endogenous stimuli.

## Discussion

The novelty of the present study is the first demonstration that high-salt exposure, through increased oxidative stress, induces UCP2 stimulation and the latter is, in turn, responsible of autophagy induction. As a result, cell viability is preserved upon the high-salt stress condition (Supplementary Fig. 3). In fact, in the absence of UCP2, autophagy is no longer activated with a significant impairment of the cell viability. To reinforce the role of autophagy as a mechanism mediating the UCP2 protection upon salt exposure, we show that an autophagy inducer, such as TAT-Beclin 1, can rescue cell viability in UCP2-silenced cells even when exposed to high-salt.

The causative role of high-salt in producing vascular and renal damage is well-known, particularly in the context of hypertension [23]. In addition, several studies have shown that high-salt favors vascular dysfunction, renal and cerebrovascular damage, independent of blood pressure levels, in the SHRSP model [3, 5, 7]. Interestingly, we previously demonstrated that high-salt diet suppressed UCP2 expression in both kidney and brain of high-salt fed SHRSP, and that upregulation of UCP2 could prevent both renal injury and stroke occurrence in this model [3–7, 11]. We concluded that a high-salt-induced oxidative stress and inflammation underlined the vascular damage of the strain and that the anti-oxidant and anti-inflammatory properties of increased UCP2 expression were the main molecular mechanisms mediating its beneficial effects toward vascular damage in the high-salt fed SHRSP.

In mice, high-salt diet has been reported to induce vascular dysfunction and this effect was again independent of blood pressure levels [9, 10]. Of note, although baseline UCP2 expression was modestly increased upon high-salt in the mouse model, it was unable to counteract the vascular damage [9, 10]. On the other hand, a relevant UCP2 upregulation, such as that observed in the transgenic mouse, could successfully prevent vascular dysfunction upon high-salt feeding [9].

An increase of oxidative stress is the common response to high-salt exposure [24]. On the other hand, it is known that increased ROS causes deleterious effects by damaging several macromolecules (DNA, lipids, proteins) finally leading to cell death [25]. For this reason, it is not surprising that increased oxidative stress may activate autophagy with the aim to clear damaged organelles and cells and to maintain a healthy cellular environment, gaining new energy and resistance to cell insults [22, 26]. In fact, autophagy is able to deliver intracellular material to lysosomes for degradation [27, 28].

Although a relationship between UCP2 and autophagy has been recently shown in other pathological contexts [13–18], a role of UCP2 in relation to autophagy upon the high-salt exposure has never been described before. In the current studies, by using endothelial and renal tubular epithelial cells, we provide the first evidence that UCP2 protects from high-salt-induced oxidative stress cellular injury through the induction of autophagy. Thus, our investigation adds more detailed mechanistic information to explain the previously described beneficial impact of UCP2 in the presence of high-salt exposure, apart from its known anti-oxidant and anti-inflammatory properties [4, 6–8].

Of note, our experimental context resembles that observed in the presence of other toxic stimuli. In fact, autophagy can be activated by other stressors like hypoxia, nutrients and growth factors deprivation, chemo- and radiotherapies [25, 29–32]. In all these conditions, accumulation of ROS is observed as a trigger for autophagy induction. Overall, these findings support the notion that autophagy is activated in the presence of increased oxidative stress as an adaptive mechanism in order to maintain a healthier environment and to preserve the tissues and organs from disease development.

Another original and interesting observation of the current study is provided by the demonstration that an interplay occurs between increased ROS, UCP2 activation and autophagy stimulation: high-salt increases ROS, consequently UCP2 expression and the autophagy machinery. However, at the highest ROS level, corresponding to that produced by UCP2 silencing, autophagy is no longer activated. In fact, the excess of ROS turns off the autophagy induction. The latter evidence is consistent with the notion that a dysregulation of redox signaling can demoralize autophagy leading to different diseases [22, 29, 30, 33]. Of note, the damaging effect of excessive ROS can be counteracted, in the absence of other cellular defenses, by an extra-stimulus of autophagy activation, such as TAT-Beclin 1. While confirming the known type of relationship between ROS levels and autophagy modulation, the present study unravels the key role of UCP2 as a mediator of this interaction in the condition of high-salt loading.

Mitochondrial ROS (mitoROS) represents the major source of oxidative stress within the cells and UCP2 acts as an important mitoROS scavenger [34, 35]. We observed a reduced mitophagy in UCP2-silenced cells, which suggests that the defective removal of damaged mitochondria may be responsible for the excess of ROS. On the other hand, high-salt exposure of UCP2-overexpressing cells led to decreased mitochondrial mass. This evidence indicates that mitophagy activation may explain the role of UCP2 in preventing ROS increase. Further studies should dissect out the fine molecular mechanisms by which UCP2 activates PARKIN and mitophagy.

Some limitations of the present work should be acknowledged. In particular, we were unable to detect, although expected, a different rate of cell death in UCP2-silenced cells exposed or not to high-salt treatment.

In conclusion, our study shows that UCP2 mediates the interaction between high-salt-induced oxidative stress and autophagy activation to preserve cell viability in vascular and renal cell lines. The key relevance of UCP2 upon high-salt exposure was revealed by the dramatic consequences of its lack, which was associated with huge amounts of intracellular ROS and with a negative impact on the autophagic cell defense for the maintenance of cell viability. We also showed that an extra-stimulus of autophagy activation, in the absence of other cellular defenses, could rescue the ROS damaging effects in UCP2-silenced cells, particularly when exposed to the high-salt stress.

Our data confirm the protective role of UCP2 and they underscore the importance of autophagy as an efficacious mechanism to counteract the high-salt-induced oxidative stress damage in both endothelial and renal tubular cells.

## Materials and methods

### Cell culture and transfection

Rat Brain Microvascular Endothelial Cells (RBMEC- -P10308; INNOPROT, Derio, Spain) and rat renal Proximal Tubular Epithelial Cells (RRPTEpiC-, P10607; INNOPROT-Derio, Spain) were cultured at 37 °C at 5 % CO_2_ and silenced for UCP2 using a UCP2-specific siRNA (SASI_Rn01_00069267, SASI_Rn_00069267_AS; Sigma Aldrich, Milan, Italy) for 24 h, following the manufacturer’s protocol. Cells incubated with transfection agents (Lipofectamine 3000 Reagent, L3000001, Invitrogen, Carlsbad, CA) were used as control cells. RBMEC and RRPTEpiC between passages 2 and 3 were used for experiments. In order to obtain UCP2 overexpression, cells were transfected with a plasmid carrying UCP2 (UCP2 Rat Tagged ORF Clone, RR204365, OriGene Technologies, Rockville, US) for 24 h, following the manufacturer’s protocol.

### Cell treatments

UCP2-silenced (siUCP2) or UCP2-overexpressing (ovUCP2) cells were used to assess the modulation of autophagy, autophagic flux, mitophagy, cell survival, apoptosis, necrosis and ROS at baseline or under stress condition (high-salt treatment). The latter was performed by treating cells for 72 h with 20 mM NaCl. The concentration and time of exposure of high-salt treatment were established following our previous published procedures in vitro [7, 36] Control cells were treated with the medium used for the routine culture. In a separate set of experiments, RBMEC were treated with increasing concentrations of H_2_O_2_ for 12 h (0.1 μM, 1 μm, 5 μM, 10 μm) for the assessment of ROS level, autophagy and autophagic flux.

### Autophagy and autophagic flux evaluation

Autophagy was evaluated by the analysis of markers of autophagy (LC3 and ATG7) by western blot experiments. For the evaluation of the autophagic flux, 100 nM bafilomycin A1 (B1793, Sigma Aldrich,) was used as an inhibitor of the autophagic flux and added to the medium 3 h before the end of the NaCl treatment.

### mRFP-GFP-LC3 tandem probe

Autophagic flux was also assessed by the mRFP-GFP-LC3 probe, as reported elsewhere [20, 37]. RBMEC were plated in 8-well chamber slides and transduced with adenovirus overexpressing mRFP-GFP-LC3 for 48 h. GFP is degraded by lysosomal acids whereas mRFP is resistant. Thus, autophagosomes were detected both in red (mRFP) and green fluorescence (GFP) whereas autophagosolysosomes were detected only in red fluorescence (mRFP). Images were randomly acquired with an epifluorescence microscope.

### Mitophagy and mitochondrial biogenesis evaluation

Mitophagy was evaluated by the analysis of marker of mitophagy (PARKIN) by western blot experiments and by the means of mitochondrial probe MitoTracker green (Invitrogen, M7514). For this purpose, 1×10^4^ cells were plated in 96 Well Black/Clear Bottom Plate and treated as described above. Afterword, cells were incubated with 100 nM MitoTracker green for 30 min at 37 °C. After three washes with PBS 1x, fluorescent signals were read with a microplate reader (excitation 490 nm, emission 516 nm). For mitochondrial biogenesis evaluation, the expression level of PGC1α was assessed by RT-PCR.

### Fluorescence-activated cell sorting (FACS) analysis

Live, apoptotic, and necrotic cells were measured by FACS by the use of a BD FACSCalibur™ Cell Analyzer (BD Biosciences, San Jose, CA) using the Annexin 5/propidium iodide staining (Annexin V-FITC ApopKit, BMS500FI-300, Invitrogen). FACS analysis was performed also in cells treated with the enhancer of autophagy TAT-Beclin D11 (NBP2-49888, Novus Biological, Centennial, Colorado, US). TAT-Beclin D11 was diluted in OPTIMEM (Invitrogen) and used at 10 μM concentration following the manufacturer’s protocol.

### ROS determination

Cellular ROS were evaluated in transfected and high-salt-treated RBMEC using the fluorescent probe 2′,7′-Dichlorofluorescein diacetate (DCFH-DA, 35845, Sigma Aldrich). Production of ROS was measured by a microplate reader (485 nM ex/535 nM em). ROS were also assessed in RBMEC treated with H_2_O_2_ for 12 h (0.1 μM, 1 μM, 5 μM, 10 μM).

### Western blot

Western blot was performed as previously described. The following primary antibodies were used: anti-LC3 (M186-3, MBL International, Woburn, MA, US), anti-UCP2 (89326, Cell Signaling Technology, Danvers, MA), anti-β-ACTIN (A5316, Sigma Aldrich), anti-ATG7 (8558, Cell Signaling), anti-PARKIN (4211, Cell Signaling), anti-VINCULIN (v4139, Sigma Aldrich). Secondary antibodies were anti-rabbit (AP132P, Millipore, Burlington, MA, US) and anti-mouse (401215, Millipore,). Proteins were detected by ECL Prime (Amersham Biosciences, CA) and acquired by a Chemidoc (Image Lab 6.0.1, Biorad, Milan, Italy). The intensity of bands was quantified by using Image J software (National Institutes of Health, Bethesda, MD).

### Real-time PCR (RT-PCR)

RNA was extracted from siUCP2 or ovUCP2 cells by TRIZOL (Invitrogen) and 500 ng were retrotranscribed into cDNA SuperScript VILO Master Mix (Invitrogen) and used for RT-PCR experiments. RT-PCR was performed using the ViiA 7 Real-Time PCR System (Applied Biosystem, Foster City, CA, USA) and SYBR Select Master Mix (4472908, Applied Biosystem,). The amount of target DNA was calculated by the comparative 2−^DDCt^ method using β-ACTIN as housekeeping gene. The following primers were used: UCP2 sense, 5’-CATTGGCCTCTACGACTCTG-3’, anti-sense, 5’ CGGACCTTTACCACATCTGTA-3’; PGC1α sense, 5’-CTCTGGGGTCAGAGGAAGAG-3’, anti-sense, 5’-CGATGACCCTCCTCACACCA-3’; β-ACTIN sense 5’ TCATGAAGTGTGACGTTGACATCCGTAAAG-3’, anti-sense 5’-CCTAGAAGCATTTGCGGTGCACGATGGAGG-3’

### Statistical analysis

All continuous variables are shown as means ± SEM. Statistical analyses were performed only when a minimum of three independent samples were acquired and the variance was assessed. Comparisons between two groups were performed using the two-tailed student’s *t*-test. Comparisons between three or more groups were performed by one-way ANOVA followed by Bonferroni post hoc test. Comparisons were considered significant for *p* < 0.05. Graph Pad Prism (Ver 8.01 GraphPad Software, Inc., La Jolla, CA, USA) statistical software was used for the statistical analysis. The number of experiments (*N*) is indicated in the figure legends.

## Supplementary information

Supplementary File

## Data Availability

The availability of data and information on the data set used and analyzed in the current study would be provided by the corresponding author upon request.
