# Supplementary material for: An interplay between UCP2 and ROS protects cells from high-salt-induced injury through autophagy stimulation
Source: Cell Death Dis. 2021 Oct 8;12(10):919. doi: 10.1038/s41419-021-04188-4 (PMC8501098; doi:10.1038/s41419-021-04188-4)
Supplement: Supplementary file 1 — Supplementary File [file 41419_2021_4188_MOESM1_ESM.docx]

**SUPPLEMENTARY FILE**

**An interplay between UCP2 and ROS protects cells from high-salt induced injury through autophagy stimulation**

Forte et al.

**
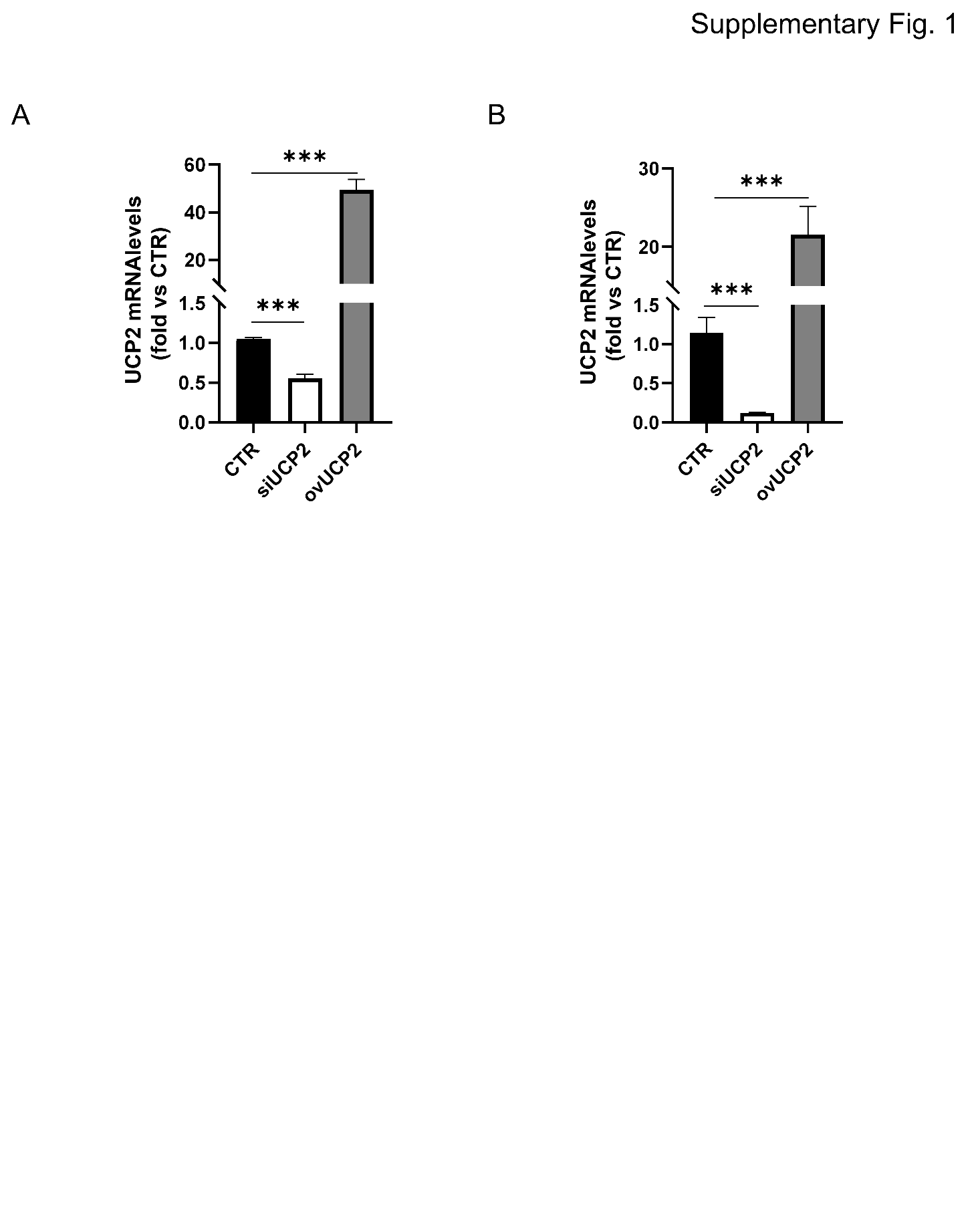
Supplementary Fig. 1. Efficacy of UCP2 gene silencing and of UCP2 overexpression.**

UCP2 mRNA levels in microvascular endothelial cells (**A**) and renal proximal tubular epithelial cells (**B**) after transfection with siRNA UCP2 (siUCP2) or with a plasmid overexpressing UCP2 (ovUCP2). (N=5). CTR indicates not transfected cells. ***p<0.001 obtained using the two-tailed students t-test. Data are reported as mean ± SEM.

**
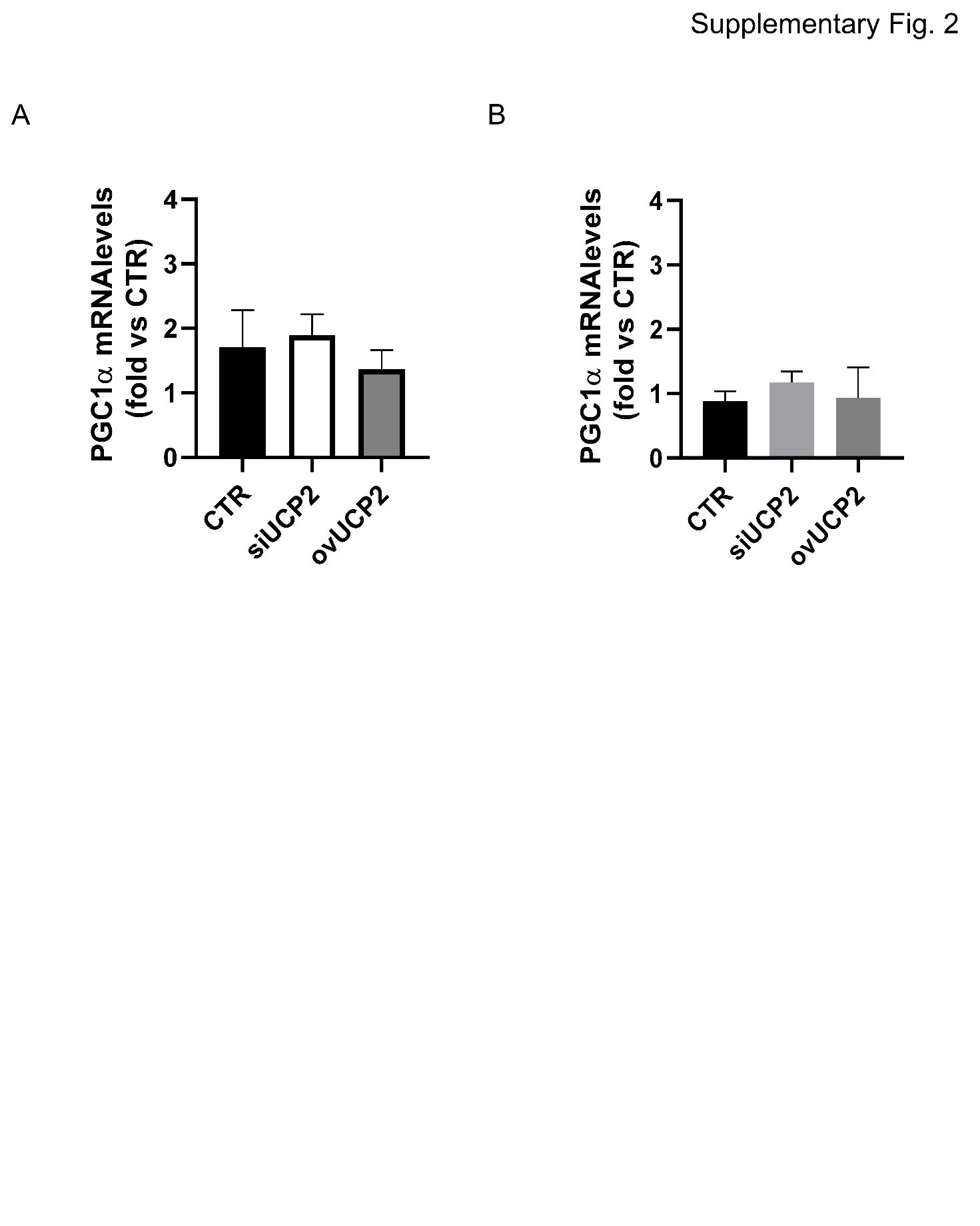
**

**Supplementary Fig. 2. PGC1α expression in UCP2 silenced cells (siUCP2) or cells overexpressing UCP2 (ovUCP2).**

PGC1α mRNA levels in microvascular endothelial cells (**A**) and renal proximal tubular epithelial cells (**B**) after transfection with siRNA UCP2 (siUCP2) or with a plasmid overexpressing UCP2 (ovUCP2). (N=4). CTR indicates not transfected cells. Differences are not significant. Data are reported as mean ± SEM.


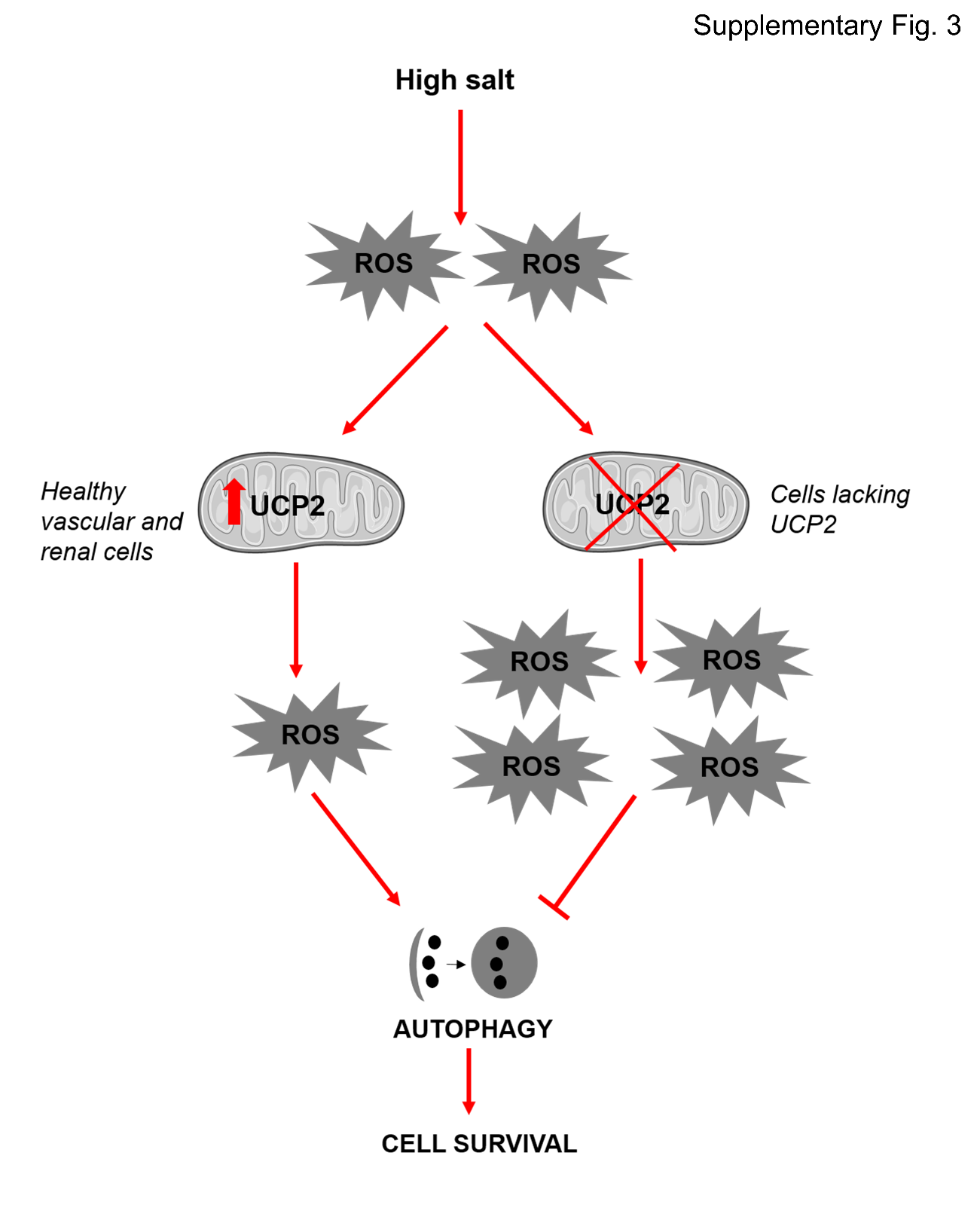


**Supplementary Fig. 3. Schematic representation and proposed mechanism of the UCP2 dependent defensive response to high-salt induced oxidative stress.** UCP2 mediates ROS reduction and autophagy stimulation to maintain cell survival upon high-salt exposure (left side). Right side: Lack of UCP2 leads to excessive ROS accumulation and impairment of autophagy.
